# Acyl-Coenzyme A: Cholesterol Acyltransferase (ACAT) in Cholesterol Metabolism: From Its Discovery to Clinical Trials and the Genomics Era

**DOI:** 10.3390/metabo11080543

**Published:** 2021-08-14

**Authors:** Qimin Hai, Jonathan D. Smith

**Affiliations:** Department of Cardiovascular & Metabolic Sciences, Cleveland Clinic, Cleveland, OH 44195, USA; haiq@ccf.org

**Keywords:** cholesterol esterification, atherosclerosis, ACAT, *SOAT*, inhibitors, clinical trial

## Abstract

The purification and cloning of the acyl-coenzyme A: cholesterol acyltransferase (ACAT) enzymes and the sterol O-acyltransferase (*SOAT*) genes has opened new areas of interest in cholesterol metabolism given their profound effects on foam cell biology and intestinal lipid absorption. The generation of mouse models deficient in *Soat1* or *Soat2* confirmed the importance of their gene products on cholesterol esterification and lipoprotein physiology. Although these studies supported clinical trials which used non-selective ACAT inhibitors, these trials did not report benefits, and one showed an increased risk. Early genetic studies have implicated common variants in both genes with human traits, including lipoprotein levels, coronary artery disease, and Alzheimer’s disease; however, modern genome-wide association studies have not replicated these associations. In contrast, the common *SOAT1* variants are most reproducibly associated with testosterone levels.

## 1. Introduction

The acyl-coenzyme A:cholesterol acyltransferase (ACAT; EC 2.3.1.26) enzyme family consists of membrane-spanning proteins, which are primarily located in the endoplasmic reticulum [1]. These enzymes act to esterify the 3-hydroxyl position of cellular free cholesterol (FC) with a fatty acid-CoA creating cholesteryl ester (CE), thus playing an important role in cholesterol homeostasis [2]. A different enzyme, lecithin cholesterol acyltransferase (LCAT), can esterify plasma FC within HDL, which is reviewed elsewhere [3]. In mammals, the ACAT family contains two isoenzymes, ACAT1 and ACAT2 [4,5,6]. ACAT1/2 are members of the membrane-bound O-acyltransferase (MBOAT) family, which includes the closely related acyl-CoA:diacylglycerol acyltransferase-1 (DGAT1) [7]. MBOAT proteins are characterized by their multiple transmembrane domains and a catalytic histidine residue in a hydrophobic domain [8].

We first deal with the confusing nomenclature of the ACAT1/2 enzymes, which are alternatively termed sterol O-acyltransferases (SOAT) and then lead to the human gene names *SOAT1* (ensemble identifier ENSG00000057252) and *SOAT2* (ENSG00000167780), whereas the mouse genes are called *Soat1* and *Soat2* [9,10]. The human *ACAT1* (ENSG00000075239) and *ACAT2* (ENSG0000012043) genes encode cytosolic enzymes with acetyl-CoA acetyltransferase/thiolase activities, which are independent from cholesterol metabolism. However, older studies may refer to the *SOAT* genes as *ACAT* genes, hampering a search of the literature. For the remainder of this article, we will refer to ACAT1/2 proteins and *SOAT1/2* genes. Given that ACAT1 is expressed more widely and robustly than ACAT2, here we will focus on ACAT1/*SOAT1*.

## 2. The Structure of ACAT1

The ACAT enzymes are large transmembrane proteins that are difficult to purify with retained activity. Therefore, purification of the ACAT enzymes is dependent on elegant cellular, molecular, and biochemical studies performed in the lab of T.Y. Chang starting in 1988, as described in his review paper [1]. Briefly, mutant Chinese hamster ovary (CHO) cells were selected that were deficient in ACAT activity and susceptible to free-cholesterol-mediated cell death [2]. These cells were transfected with human genomic DNA to isolate a clonal cell population with restored ACAT activity [11]. A cDNA library prepared from the ACAT-restored CHO line was used to make a new ACAT-restored CHO cell line leading to the molecular cloning of a human *SOAT1* cDNA, the 4.0 kb “K1 cDNA” with a 1.7 kb open reading frame [12]. Using fluorescence in situ hybridization and Southern blot analyses of human/hamster somatic cell hybrid panels, the *SOAT1* gene was mapped to the human chromosome 1q25 [13]. Then, Chang’s lab prepared an expression plasmid adding a his-tag at the N-terminus of the *SOAT1* cDNA, and additionally made monoclonal antibodies to another recombinant ACAT1 fragment [14]. The his-tagged ACAT1 was stably transfected into ACAT-deficient CHO cells and an optimized cell-free assay for ACAT activity was developed, leading to the initial purification of recombinant ACAT1 whose CE product is formed at increasing FC concentration in a sigmoidal manner, indicating the potential for catalytic and allosteric interactions with FC [14]. Among several other sterols, FC was the most potent substrate and activator of ACAT1 [15]. The steroid hormone precursor pregnenolone, made in the mitochondria, is also an ACAT1 substrate, but it cannot activate cholesterol esterification, implying that pregnenolone only binds to the catalytic site and not the allosteric site, whereas FC binds to both sites and can activate pregnenolone esterification [16]. Using recombinant ACAT1, oleoyl-CoA (18:1) vs. stearoyl-CoA (18:0) is the preferred substrate for ACAT1, leading to augmented CE production and demonstrating specific and saturable binding to ACAT1 [17]. Among unsaturated fatty acids, the ACAT1 preferentially uses oleoyl-CoA (18:1) vs. linolenoyl CoA (18:3), arachidonyl CoA (20:4), and eicosapentaenoyl CoA 20:5 [18]. The cholesterol vs. sitosterol substrate specificity of ACAT1 was compared to ACAT2 and LCAT. ACAT2 was highly selective for cholesterol, whereas ACAT1 and LCAT only slightly preferred cholesterol vs. sitosterol [19].

ACAT1 consists of two homodimers (dimer of dimers). ACAT1 contains nine transmembrane domains (TMD) and various site-directed mutagenesis studies have identified the catalytic His460 residue involved in FC binding in TMD7, and a portion of the N-terminal domain and TMD8 as being important for subunit interactions [1]. The cryo-electron microscopy structure of human ACAT1 was recently described, confirming the dimer of dimers structure and revealing three intra-ER helixes and one extra-ER helix in addition to the nine TMD-helixes [20]. Oleoyl-CoA gains entry via the C-tunnel in the structure and was found in the active site of the purified enzyme; by altering two residues in the C-tunnel, the enzyme activity was lost and the oleoyl-CoA level in the enzyme was decreased [20]. The structure demonstrates why oleoyl-CoA that bends at the Δ9 double bond fits better than nonsaturated or other unsaturated fatty acyl-CoAs; amino acid substitutions in the vicinity of the oleoyl tail also deceased activity of the enzyme [20]. The T-tunnel that converges with the C-tunnel in the active site may be used for the entry of FC [20].

## 3. The Role of ACAT in Lipid Metabolism and Atherosclerosis Derived from Knockout Mouse Studies

Both ACAT1/2 can convert cellular FC and free fatty acid to CE, which is an inert form that can be stored in lipid droplets. ACAT1 is expressed in many tissues, including macrophages and adrenal glands, and macrophage ACAT1 was thought to play a role in the formation of foam cells in atherosclerotic plaques [21]. Farese and colleagues disrupted the mouse *Soat1* gene by homologous recombination, which resulted in decreased cholesterol esterification in the *Soat1*^−/−^ embryonic fibroblasts and adrenal lysates, and markedly reduced CE levels in the adrenal glands and in peritoneal macrophages incubated with acetylated LDL. In contrast, the livers of *Soat1*^−/−^ mice contained substantial amounts of CE and exhibited no reduction in cholesterol esterification activity. The tissue-specific reductions in cholesterol esterification provided strong evidence that the mammalian intracellular CE formation involves more than one ACAT enzyme, promoting the discovery of ACAT2/*SOAT2*. *Soat1*^−/−^ mice also had higher plasma total cholesterol (TC) and high-density lipoprotein cholesterol (HDL-C) vs. controls when mice were fed either a chow or high-fat diet [21]. These global *Soat1*^−/−^ mice were also characterized to have leukocytosis and an increase in bone marrow cell proliferation [22]. Later, ACAT2 was cloned and knockout of *Soat2* in mice resulted in reduced CE synthesis in the small intestine and liver, which, in turn, limited intestinal cholesterol absorption, hepatic cholesterol gallstone formation, and the accumulation of CE in plasma lipoproteins [23,24].

The role of *Soat1* in atherosclerosis is controversial. Farese, along with collaborators Linton and Fazio, bred *Soat1*^−/−^ mice to *Ldlr*^−/−^ and *Apoe*^−/−^ mouse models of hyperlipidemia. On the *Apoe*^−/−^ background, *Soat1* deficiency led to skin xanthomas even on a chow diet; on a Western-type diet, plasma total cholesterol was markedly reduced in the *Soat1*^−/−^ mice [25]. Skin xanthomas and decreased plasma cholesterol were also observed in the *Ldlr*^−/−^ with *Soat1*-deficient mice upon feeding of a synthetic diet with 1.25% cholesterol and 0.5% cholate. These skin xanthomas were attributed to a macrophage *Soat1* deficiency via bone marrow transplantation (BMT) studies [25]. To determine if the macrophage *Soat1* alters atherosclerosis without an effect on plasma cholesterol, Fazio and Linton performed BMT from WT and *Soat1*^−/−^ donor mice into *Ldlr*^−/−^ host mice, and found larger aortic root and surface lesions in the mice receiving the *Soat1*^−/−^ marrow [26]. In contrast, Ishibashi and colleagues found smaller aortic root lesions with less CE content in *Soat1*^−/−^ vs. *Soat1*^+/+^ mice bred onto the *Apoe*- or *Ldlr*-deficient backgrounds, despite observing a lower plasma cholesterol only in the Western-type diet fed *Apoe*^−/−^ mice, and not in the *Ldlr*^−/−^ mice fed a diet with 1.25% cholesterol and 0.5% cholate [27].

Chang and colleagues bred *Soat1* floxed mice and crossed them with LysM-Cre mice to generate myeloid-specific *Soat1*-deficient mice (*Soat*^−*M*/−*M*^), which did not have leukocytosis as seen in the global KO mice [28]. Upon crossing these mice with *Apoe*^−/−^ mice, they found significantly decreased aortic and aortic root lesions vs. *Apoe*^−/−^ control mice accompanied by decreased lesion macrophages, macrophage recruitment, and macrophage β1-integrin; however, these mice developed larger skin xanthomas [28]. These findings were extended to advanced lesions, where decreased cholesterol clefts were seen in the *Soat1^−M/−M^Apoe*^−/−^ mice [29].

Farese and colleagues then examined the contribution of *Soat2*-derived CE to atherosclerosis by crossing *Soat2*^−/−^ mice with Apoe-deficient mice [30]. *Soat2*^−/−^ *Apoe*^−/−^ mice and *Soat2^+/+^ Apoe*^−/−^ control mice showed a similar increase in plasma apoB and total plasma lipids; however, the lipid content of the apoB-containing lipoproteins in the double-null mice contained primarily triglycerides rather than CE. At 30 weeks of age, the *Soat2*^−/−^ *Apoe*^−/−^ mice fed with a chow diet had smaller atherosclerosis lesions in all aortic regions (arch, thorax, and abdomen) than the *Soat2^+/+^ Apoe*^−/−^ controls. These investigators surmised that the reason for smaller lesions in the *Soat2*^−/−^ *Apoe*^−/−^ mice was due to high triglycerides but low CE levels in plasma. These findings highlight the crucial role of *Soat2*-mediated effects on plasma CE and atherosclerosis in mice, supporting the therapeutic potential for *SOAT2* inhibition.

## 4. ACAT Inhibitors in Pre-Clinical Studies

Due to their role in cholesterol metabolism, ACAT enzymes became a logical target for pharmacological inhibition to protect against atherosclerosis. Since ACAT1 is responsible for CE accumulation in macrophage foam cells, its inhibition was thought to reduce cholesterol accumulation in atherosclerotic lesions. Conversely, intestinal and liver ACAT2 plays an important role in cholesterol absorption; thus, its inhibition was thought to lead to reduced cholesterol absorption and serum levels. Therefore, many preclinical studies have been performed using various ACAT inhibitors, as demonstrated by the select examples below. These and additional examples are shown in Table 1.

- Rabbit models

In 1991, Krause and colleagues used the Parke-Davis ACAT inhibitor CI-976 in New Zealand white rabbits being fed a high cholesterol diet and reported an increased regression of pre-existing lesions and a reduced formation of new lesions [49]. A different ACAT inhibitor, avasimibe (CI-1011), when given after changing from a high cholesterol to a normal chow diet, was also found to reduce pre-existing lesion cholesterol and macrophage content in rabbits, despite having no significant effects on plasma cholesterol levels [39]. Similar findings in rabbit studies have been observed with several other ACAT inhibitors, such as NTE-122 and F-1394 [58,59].

- Mouse models

Avasimibe was found to lower plasma cholesterol levels in hypercholesterolemic apoE*3-Leiden mice fed a high-fat, 0.5% cholesterol, 0.1% cholate diet, leading to reductions in the aortic root lesion area, lesion severity, and monocyte adherence to the aortic root endothelium [41]. Fisher and colleagues demonstrated that the ACAT inhibitor F-1394 led to a dose-dependent decrease in the atherosclerotic lesion area and macrophage content, accompanied by a decrease in total plasma cholesterol in *Apoe*^−/−^ mice fed a Western-type diet [34]. These results were extended to pre-existing lesions, where the mice treated with an ACAT inhibitor had slower lesion progression, with less macrophage, FC, and CE accumulation [35]. After switching *Apoe*^−/−^ mice from a Western-type diet to a chow diet, the short-term addition of F-1394 decreased the lesion macrophage pro-inflammatory gene expression and increased the inflammation resolving gene expression [36]. Pactimibe sulfate (CS-505) treatment of *Apoe*^−/−^ mice fed a chow diet decreased plasma cholesterol along with aortic root lesion development and progression, with the lesions being more significantly reduced with pactimibe sulfate vs. avasimibe treatment, despite similar effects on plasma cholesterol [43]. This compound was later used in human clinical trials (see below).

- Adverse effects of ACAT inhibitors

The ACAT inhibitor PD 132301-2 was found to have adrenal toxicity in guinea pigs and dogs, leading to decreased plasma cortisol at baseline and after ACTH stimulation, and was associated with decreased adrenal cholesterol levels and increased release of liver enzymes into plasma [60,61]. Ex vivo adrenocortical cells treated with PD 132301-2 showed decreased mitochondrial function and ATP levels that preceded cell death [62].

Rothblat and colleagues found that an ACAT inhibitor treatment of cholesterol-loaded mouse macrophages led to increased cell death due to the buildup of free cholesterol [63,64]. Later, this group demonstrated that ACAT inhibition in cholesterol-loaded mouse macrophages led to intracellular FC crystal formation, and release of these crystals from the cells which continued to grow in size [65]. Tabas and colleagues published a series of elegant papers which examined the mechanism of macrophage cell death by FC loading via incubation with acetylated LDL in the presence of an ACAT inhibitor. The cell death was attributed to apoptosis, as it was blocked by caspase inhibitors, and, macrophages from the mice with a dysfunctional Fas ligand or Fas receptor were resistant to FC-loading-induced apoptosis [66]. In addition to the Fas pathway, they also showed that macrophage FC loading decreased the mitochondrial membrane potential leading to increased Bax levels and mitochondrial cytochrome C release, which resulted in caspase activation and apoptotic cell death [67]. More details were elucidated about the signaling involved in ACAT-inhibitor-mediated FC macrophage apoptosis. FC loading in macrophages activates the unfolded protein response, leading to the induction of the CHOP protein, and this induction requires the p38 MAP kinase activation cascade [68]. In addition, ligation of the scavenger receptor A (which mediates uptake of acetylated LDL) and JNK2 kinase are also necessary for FC-loading-induced macrophage apoptosis [68].

## 5. Clinical Trials of ACAT Inhibitors

Animal studies of ACAT inhibitors yielded sufficient evidence of anti-atherosclerosis activity that three human clinical trials were published using two different ACAT inhibitors. In a multi-center, randomized, double-blind, placebo-controlled clinical trial, placebo or three dose of avasimibe, along with lipid-lowering therapy, were administered to 639 coronary artery disease patients to examine changes in coronary atheroma volumes measured by intravascular ultrasound (IVUS) [69]. Compared to baseline atheroma volumes, there was no significant benefit of avasimibe after 24 months of treatment [69]. Additionally, the treated patients had increased LDL-C vs. baseline values.

Similarly, in the ACTIVATE trial, Nissen and colleagues enrolled 408 patients with confirmed coronary artery disease, in a multi-center, randomized, double-blind, placebo-controlled clinical trial of the ACAT inhibitor pactimibe [70]. Eighteen months of treatment vs. baseline failed to show a significant improvement in atheroma volume assessed by IVUS. Additionally, the treated vs. placebo patients had significantly less atheroma regression in the most diseased artery segment [70].

The CAPTIVATE trial was another multi-center, randomized, double-blind, placebo-controlled clinical trial which used the ACAT inhibitor pactimibe in 892 patients with heterozygous familial hypercholesterolemia, to assess carotid artery intimal-media thickness at baseline and at 12, 18 and 24 months later [71]. This trial was terminated prematurely after an average follow-up of 15 months when the results of the ACTIVATE trial became available. LDL-C increased by 7.3% in the pactimibe group, which was significantly more than the 1.4% increase in the placebo group. More importantly, major cardiovascular events were significantly increased in the treated vs. placebo patients [71].

There has been speculation about why ACAT inhibitors were successful in some animal models but not in human clinical trials. Global ACAT inhibition may lead to excess free cholesterol that can lead to cytotoxicity and proinflammatory effects that may not be beneficial for atherosclerosis, leading to the suggestion that ACAT2 selective inhibition might overcome this [72]. Avasimibe may have off-target effects that are also contraindicated, such as the activation of the human pregnane X receptor and the induction of CYP3A4, CYP2C9, and CYP2B6 among other genes [73,74], which may increase the catabolism of protective drugs, such as statins.

## 6. ACAT Genetics and Genomics

### 6.1. Mouse Soat1 Genetics

Adult male AKR mice have adrenocortical lipid depletion [75]. Farese and colleagues showed that this was due to a deletion of the first coding exon of the *Soat1* gene, leading to an N-terminally truncated ACAT1 protein; however, ACAT enzyme activity was still present in the adrenal membranes and transfected CHO cells [76]. Using classical mouse genetic methods combined with CRISPR-Cas9 gene editing, our lab independently identified that the mouse *Soat1* gene was responsible for macrophage CE levels after cholesterol loading, with the AKR allele having an exon 2 deletion leading to a lower CE accumulation [77]. Furthermore, the AKR decrease in macrophage ACAT activity can lead to more FC accumulation that can induce CHOP protein expression by acetylated LDL loading in the absence of an ACAT inhibitor [78].

### 6.2. Human SOAT1/2 Genes and Expression

Searching for the human *SOAT1* gene using the UCSC genome browser (http://genome.ucsc.edu, accessed on 30 July 2021) shows that its 16 exons reside on chromosome 1 from 179,293,718–179,358,679 bp (GRCh38). Chang’s lab previously reported four human *SOAT1* mRNAs on Northern blots [79]. Additionally, one of the mRNAs contained two additional exons, a 1277 bp exon derived from chromosome 7, called exon X_a_ that is not conserved in mice, and a second 10 bp exon that was from an unknown location, called exon X_b_ [79]. Our analysis of the exon X_a_ sequence places it at 120,669,474–120,670,751 (GRCh38—strand) within an intron of the KCND2 gene. Thus, this mRNA was thought to be due to trans-splicing that was confirmed by using human RNA with RNAse protection and cDNA PCR assays [79]. In transfection studies, another *SOAT1* isoform was characterized that contains not only sequences from chromosomes 1 and 7, but also a sequence derived from the antisense strand of a plasmid-derived ampicillin resistance gene, claiming an exogenous–endogenous trans-splicing system [80]. The existence of trans-splicing in human mRNAs is controversial, as generation of cDNA libraries can lead to many artifactual chimeric mRNAs [81,82]. To provide a fresh look at this, we searched the GTEx project (gtexportal.org) and identified the adrenal gland, cultured fibroblasts, lung, EBV transformed lymphocytes, prostate, cervix, and spleen as the human tissues with the highest expression of *SOAT1*. Within the GTEx portal, we searched RNAseq reads in these tissues through the IGV Browser and found abundant reads in the *SOAT1* exons on chromosome 1, but no reads through the Exon X_a_ on chromosome 7. Thus, we found no convincing RNAseq evidence for this chimeric transcript in human tissue.

The human *SOAT2* gene contains 15 exons and maps to chromosome 12q13 from 53,103,485–53,124,534 bp (GRCh38). The GTEx portal shows the highest expression in the human spleen, small intestine, tibial nerve, aorta, and cervix; and surprisingly, the liver is listed as the 10^th^ highest expressing tissue.

### 6.3. Human SOAT1/2 Common and Rare Genetic Variants and Disease Association

Before the era of genome-wide association studies (GWAS), researchers studied one or several common variants for association with a trait in relatively small studies involving humans, using *p* < 0.05 as the threshold for significance, which has yielded many false positive results [83]. There have been multiple studies that include subjects of Asian, European, and African descent reporting a single nucleotide polymorphism (SNP) in or near the *SOAT1* or *SOAT2* gene to be associated with lipoprotein levels, incidence of atherosclerotic disease, or Chagas disease, some of which were sex-specific or population specific [84,85,86,87,88,89,90,91,92,93]. In a well-powered meta-analysis, the *SOAT1* SNP rs4421551 was associated with HDL-C (*p* = 4.81 × 10^−3^, *n* = 7840 subjects) [94]. There have also been *SOAT1* variants that are associated with Alzheimer’s disease [95,96]. Knockout of mouse *Soat1* or ACAT1 inhibitors has been found to decrease β-amyloid levels in mouse models of Alzheimer’s disease [97,98,99,100].

In the modern GWAS era, the threshold for genetic association significance is much more stringent at *p* < 5 × 10^−8^. Searching the GWAS catalog in July 2021 found only seven associations for SNPs nearest to the *SOAT1* gene, four of which are for testosterone levels, and one each for morningness, type 2 diabetes, and white matter microstructure (Table 2). There are abundant *SOAT1* expression quantitative trait locus (eQTL) SNPs associated with *SOAT1* expression in many tissues in the GTEx portal. The finding of no *SOAT1* GWAS SNPs associated with lipoprotein levels, atherosclerotic disease, or Alzheimer’s disease, which have been the objectives of large, well-powered GWAS studies, indicates that the *SOAT1* expression levels do not play a significant role in these phenotypes. Of the three SNPs associated with testosterone levels, two (rs2152318 and rs2248979) were strong eQTL SNPs associated with *SOAT1* expression in the adrenal gland. This may be biologically plausible, as adrenal steroids can be converted to androgens [101]. Likewise, searching the GWAS catalog for *SOAT2* identified only one association for age-related macular degeneration, although this failed to meet the stringent GWAS *p*-value threshold (Table 2). Again, numerous eQTL SNPs associated with *SOAT2* expression are found in the GTEx portal; however, not in the small intestine or liver, the two tissues that might affect lipid metabolism the most.

One limitation of GWAS is that they are underpowered for rare variants, whose discovery is mainly through exome and whole genome sequencing. We searched the ClinVar database (ncbi.nlm.nih.gov/clinvar/, accessed on 30 July 2021) for rare *SOAT1/2* variants in July 2021. *SOAT1* rare variants in the coding region include four synonymous (all classified as benign) and one non-synonymous variant (rs73048613, Gln204GLu), the latter of which is enriched in African populations (~4%) and is very rarely found in European populations, but is classified as benign by computational analysis and by one submitter. *SOAT2* rare variants in the coding region include two synonymous (both classified as benign) and one non-synonymous variant (rs149485045, Arg417Gln), the later which has a <1% allele frequency in all populations, but also being classified as benign. In summary, neither common nor rare variants in *SOAT1/2* are associated with lipoprotein levels, atherosclerosis, or Alzheimer’s disease, despite abundant biochemical and mouse studies showing effects on related phenotypes. Thus, a role for selective ACAT2 inhibition as a human therapeutic is not supported by human genetic studies.

## 7. Conclusions

ACAT enzymes play essential roles in the storage of cellular cholesterol as cholesteryl esters, which help to protect cells from the adverse effects of excess free cholesterol. Long-standing research projects, including mouse gene knockout studies, have led to understanding the tissue expression differences and the roles of ACAT1 and ACAT2 in lipoprotein metabolism, adrenal function, and cholesterol absorption, which supported preclinical and clinical trials of ACAT inhibitors. Clinical trials using non-selective ACAT inhibitors have not proven to be effective in reducing atheroma, and can lead to a small increase in LDL-C in familial hypercholesterolemia heterozygous patients. In addition, there is little support for ACAT enzymes as a drug target for cardiovascular disease based on the lack of findings from *SOAT1* and *SOAT2* genome-wide association studies, and the absence of rare variants associated with relevant phenotypes.

## Figures and Tables

**Table 1:** Pre-clinical studies of the main ACAT inhibitors for atherosclerosis.

Name	Animal	Plasma Cholesterol	Atherosclerotic Lesion Effect	Other Effects	Reference
F-1394	Rats	↓	N.D.	↓ cholesterol absorption	[31]
	Apoe^−/−^ ldlr^−/−^ mice	N.S.	↓		[32]
	Rabbits (balloon injury)	N.S.	N.D.	↓ neointimal thickening	[33]
	Apoe^−/−^ mice	↓	↓		[34]
	Apoe^−/−^ mice	N.S.	↓ progression	↓ lesion tissue factor	[35]
	Apoe^−/−^ mice	N.S.	N.S.		[36]
	Apoe^−/−^ and human APO A1-transgenic mice	N.S.	N.S.		[36]
CI-1011 (avasimibe)	Male monkeys	↓	N.D.	↓ Lp(a)	[37]
	Miniature pigs	↓	N.D.		[38]
	Male rabbits	N.S.	↓ progression	↓ lesion MMP expression	[39]
	Beagle dogs	N.D.	N.D.	↑ Emesis, saliva, hepatic toxicity,	[40]
	ApoE *3-Leiden mice	↓	↓		[41]
	Miniature pigs	↓	N.D.		[42]
	Apoe^−/−^ mice	↓	↓ progression		[43]
	Rabbits	N.D.	↓ progression		[44]
CS-505 (pactimibe sulfate)	Apoe^−/−^ mice	↓	↓ progression	↓ MMP expression	[43]
	Rabbits	N.S.	N.S.	↑ lesion fibers and smooth muscle cells	[45]
	Hamsters	↓	↓		[46]
	Hamsters	↓	N.D.	↓ hepatic lipids; ↓ lipid absorption; ↑ fecal lipid excretion	[47]
	Monkeys	↓	N.D.		[47]
	Diabetic rats	N.D.	N.D.	↓ postprandial fat loading	[47]
	Apoe^−/−^ mice	↓	↓		[48]
CI-976	Rabbits	N.S.	↓ progression		[49]
	Hamsters	↓	N.D.		[50]
	Rats	↓	N.D.	↓ liver CE	[51]
	Micropigs	N.S.	N.D.	↓ liver CE	[52]
	Endogenous hypercholesterolemia rabbits	↓	N.D.		[53]
	Rabbits	↓	↓		[54]
	Nephrotic syndrome (NS) rats	↓	N.D.	restore LDL and HDL receptors	[55]
K-604 (ACAT-1 selective)	Hamsters	↓ (at high dose)	↓		[56]
	Apoe^−/−^ mice	↓ (at high dose)	N.S.	↑ lesion collagen	[48]
PD 132301-2	Rats	↓	N.D.		[57]
	Guinea pigs	↓	N.D.		[57]
	Rabbits	↓	N.D.		[57]
CL 277,082	Rats	↓	N.D.		[57]
	Guinea pigs	↓ (at high dose)	N.D.		[57]
	Rabbits	↓	N.D.		[57]
	Rats	↓	N.D.		[51]

N.D., not determined; N.S., not significantly different; MMP, matrix metalloproteinases.

**Table 2:** GWAS findings for human *SOAT1/2* genes.

Gene	Variant	*p*-Value	Reported Trait	Reference
*SOAT1*	rs13306728	6 × 10^−14^	Morningness	[102]
	rs2152318	2 × 10^−27^	Bioavailable testosterone levels	[103]
	rs2248979	2 × 10^−12^	Bioavailable testosterone levels	[103]
	rs569421885	1 × 10^−62^	Total testosterone levels	[103]
	rs569421885	3 × 10^−37^	Total testosterone levels	[103]
	rs2816177	7 × 10^−9^	Type 2 diabetes	[104]
	rs67563284	2 × 10^−8^	White matter microstructure	[105]
*SOAT2*	rs11170417	7 × 10^−6^	Age-related macular degeneration progression	[106]
